# Designing and implementing a metacognitive model in healthcare simulation

**DOI:** 10.3389/fmed.2026.1934102

**Published:** 2026-09-23

**Authors:** Helen R. Church, Emma Howie, John Sandars

**Affiliations:** 1 Medical Education Centre, University of Nottingham, Nottingham, United Kingdom; 2 Surgical Sabermetrics Laboratory, The University of Edinburgh, Edinburgh, United Kingdom; 3 School of Medicine, Edge Hill University, Ormskirk, United Kingdom

**Keywords:** health professions education, metacognitive coaching, metacognition, self-regulated learning, simulation based education

## Abstract

Simulation-based education (SBE) is an evidence-based and widely used pedagogical approach for acquiring a range of clinical skills. However, because much of SBE takes place within isolated and controlled environments, there are limitations regarding its transfer to real-world clinical practice. While considerations such as fidelity and multidisciplinary involvement can enhance the transferability of learning outcomes to clinical settings, the cognitive and emotional demands of clinical practice, which are known to influence performance, are often overlooked. To optimise transferability from simulation to bedside care, learners need to develop their metacognitive ability to monitor and regulate their cognitive and emotional processes to the demands of practice. In this article, we highlight the importance of metacognition and share insights from the development of a conceptual metacognitive model and its implementation within SBE.

## Introduction

Simulation Based Education (SBE) often takes place within isolated and controlled environments, and this limits its transfer to real-world clinical practice (1) Transferability can be increased by considering fidelity and multidisciplinary involvement, but the important cognitive and emotional demands of clinical practice, which are known to influence performance, are often overlooked. To optimise transferability from simulation to bedside care, learners need to develop their ability to adapt their cognitive and emotional processes to the demands of practice. This requires SBE to provide opportunities for enhancing learners' metacognition, with the ability to actively monitor and regulate their own cognitive and emotional processes both during SBE and in real-life clinical practice.

## Theoretical foundations of metacognitive models

Metacognition is a higher-level mental process and was originally described as “thinking about thinking” by Flavell (2). This description was later expanded by Nelson and Narens (3) through their influential two-level model, consisting of the object level and the meta level. The object level represents cognitive processes, such when using strategies for clinical reasoning, decision-making, and performing clinical procedures, whereas the meta level involves monitoring and regulating these cognitive processes. Two key metacognitive processes connect these levels: monitoring, where learners assess their understanding and performance, and control, where they modify cognitive processes in response to those assessments. There has been widening of the definition to include a broader range of metacognitive processes, including planning (deciding how to approach a task), monitoring (checking understanding and progress while performing), evaluating (judging the effectiveness of one's approach or solution) and adapting strategies based on the performance of the task) (4).

Simulation provides an ideal environment for learners to actively use and develop their metacognitive processes, with learners having to continuously monitor their performance across a variety of clinical tasks and control their cognitive processes during evolving scenarios (5). Debriefing further strengthens monitoring and control by encouraging reflection on the accuracy of judgments and the effectiveness of actions (5).

Several commonly applied educational theories in simulation-based education place metacognition at their core. Kolb's Experiential Learning Theory (6) proposes that learning occurs through a cycle of concrete experience, reflective observation, abstract conceptualization, and active experimentation. Simulation provides realistic experiences, while metacognitive reflection and debriefing allow learners to analyse their actions and apply lessons learned to future situations. Zimmerman's Self-Regulated Learning Theory (7) highlights learners' active role in setting goals, monitoring progress, and evaluating outcomes. Simulation activities promote these regulation processes by encouraging learners to assess their performance, respond to metacognitive feedback, and refine strategies. Schön's Reflective Practice Theory (8) further highlights the importance of reflection-in-action and reflection-on-action. During simulation scenarios, learners monitor and adjust their thinking in real time, while post-simulation debriefing enables deeper metacognitive reflection on decisions and outcomes. These reflective processes strengthen professional judgment and self-awareness. Importantly, metacognition enhances transferability from simulation to bedside care by enabling learners to monitor and regulate their cognitive processes across different contexts (9).

The traditional view of metacognition with its focus on cognitive processes has been broadened by Efklides' Metacognitive and Affective Model of Self-Regulated Learning (10) by incorporating emotional and motivational factors. Learners monitor not only their cognitive processes but also their confidence, anxiety, effort, and emotional responses. In simulation settings, where stressful and uncertain situations are often recreated, emotional awareness of ‘metacognitive feelings’ or ‘metacognitive behaviours’ supports the development of self-regulation and adaptive expertise across different contexts (11).

## Development of a conceptual model

Simulation provides an appropriate environment in which learners can develop and rehearse the application of metacognitive processes before transferring these skills to clinical practice. To facilitate this transfer, a structured framework, or *conceptual model*, was developed to guide the coaching and application of metacognition within simulation-based learning.

Tappenden et al. (12), define a conceptual model as “*the abstraction and **representation** of **complex phenomena** of interest in some **readily expressible form**, such that individual stakeholders’ **understanding** of the parts of the actual system, and the mathematical representation of that system, may be **shared, questioned, tested,** and **ultimately agreed**”.*

This definition clearly describes the development of the conceptual model which became known as ‘PERFORM’ (Performance Enhancing Routines For Optimising Readiness using Metacognition). The focus of this metacognitive model was on monitoring and regulating the application of the learner's preferred Performance Enhancing Routines (PERs), such as visualisation, during a clinical task. The complexities of the different psychological ‘domains’ of metacognition (metacognitive- feeling/behaviour, knowledge, experience, and regulation), which are often unfamiliar to clinicians, can be represented visually (see Figure 1).

**Figure 1:**
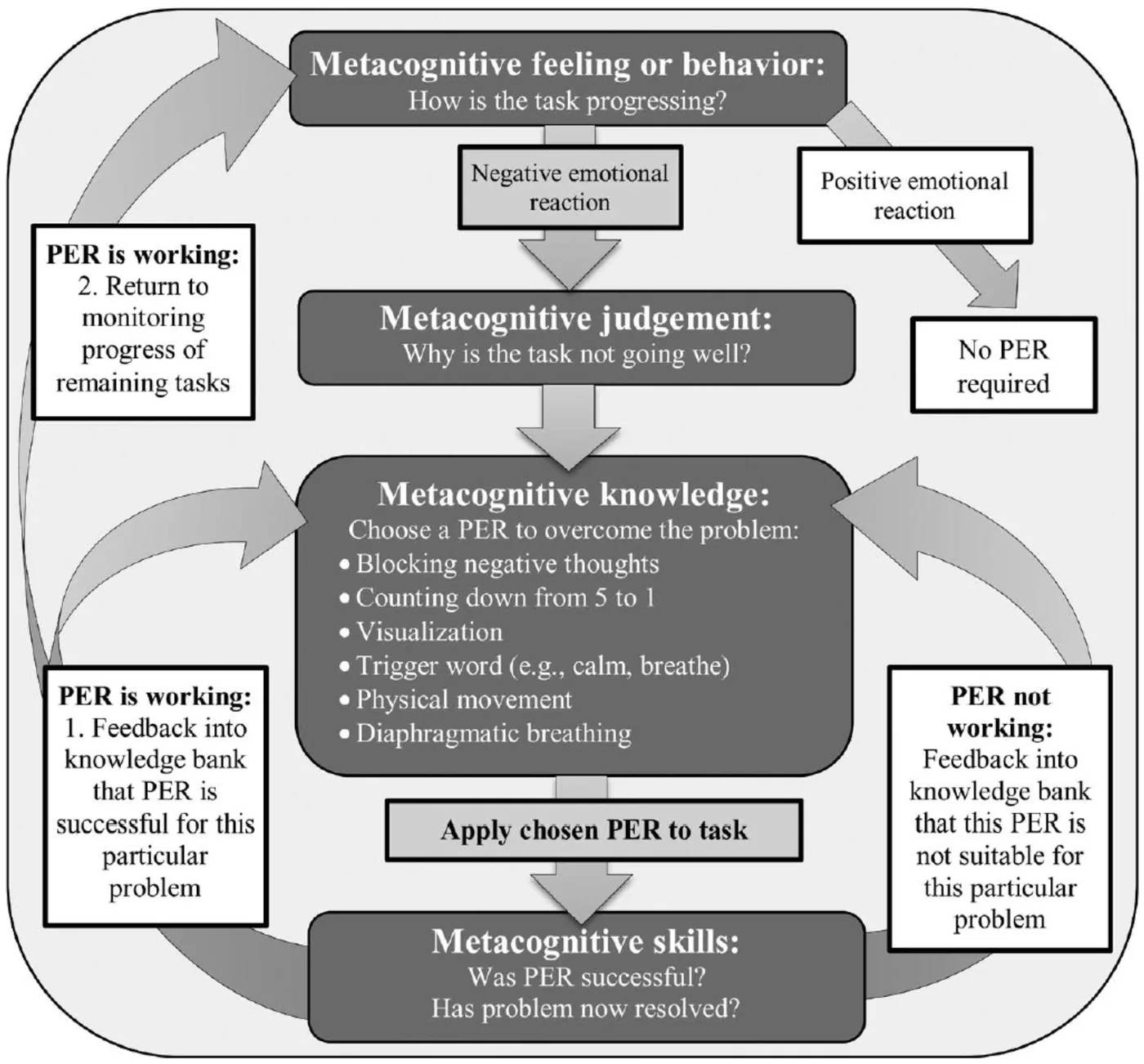
PERFORM model. Reprinted with permissions (13).

Taking the domains, as described by Efklides (14), and arranging them into a flow diagram enabled them to be more easily understood, instructional and coachable to stakeholders (in this case, newly qualified doctors as study participants). This shared vision was achieved by providing examples taken directly from Efklides' description of domains using ‘every-day’ tasks (e.g., reading a book). This enabled participants to recognise their, albeit unknowing, existing knowledge of what a metacognitive feeling (e.g., under-confidence, anxiety) or behaviour (e.g., tremor, fidgeting) ‘feels’ like. Once established, this was then contextualised into the clinical setting by talking through the model using common clinical challenges (e.g., acute scenarios) reinforced their understanding of the model, and how it might apply in clinical practice.

## Proof of concept: supporting foundation doctors in managing acute illness

Referring back to the above definition, ‘testing’ the model was done through a proof-of-concept study with newly qualified doctors (within 2 years of graduation). After receiving coaching in the use of the PERFORM model, the doctors applied it during patient encounters over a three-month period, completing a reflective diary and participating in a follow-up interview after each event. The model was tested and individualised (through the selection of Performance Enhancing Routines listed in the Metacognitive Knowledge box in Figure 1) across a range of scenarios and contexts and, owing to its generalisability beyond the clinical environment, it was also applied in non-clinical tasks and personal life events.

An *in-situ* simulation was conducted at the end of the study, with full audio-visual recording of the scenario. Participants viewed this recording using a think-aloud protocol immediately at the end of the simulation, verbalising their planning, monitoring, and regulation of actions as they progressed through the task, with scaffolds or prompts to encourage self-narration, where needed, e.g., “*tell me what's going on here*…”, “*what are you thinking or feeling in this part of the scenario*…”. The major outcomes of the study were that the model was successful in helping to control negative effects of emotions and behaviours triggered by acute patient scenarios, and (of equal importance) the conceptual model had practical utility, with real-world applicability.

### Adapting PERFORM across contexts: tailoring and contextualisation

The conceptual design of the PERFORM model makes it easily transferable between different settings and for different seniority levels of participants, as evidenced in the Surgical Utilisation of Performance Enhancing Routines (SUPER) study (15). This transferred the PERFORM model into a surgical setting, retaining the original domains and their sequence, whilst adapting the teaching and examples to reflect the surgical context. The complexity of the scenarios was also pitched at an appropriate level for the resident doctors' level of training. The original Performance Enhancing Routines (PER's) were also selected and adapted based on the potential usability within surgery, for example, visualisation of the surgical procedure.

The aim of the SUPER study was to investigate the effect of the PERFORM model on surgical performance, specifically on technical skills and perceived workload. The SUPER study was a randomised pilot in which 29 surgically interested resident doctors (below ST3 level) completed standardised laparoscopic simulation tasks pre- and post- intervention. The intervention group received an hour of teaching relating to the PERFORM model in surgical settings. The PERFORM group improved on blinded technical skill ratings while the control group did not, with the clearest gains in efficiency and tissue handling. Overall subjective workload fell further in the intervention group, and situational stress dropped with a large effect. The metacognitive intervention changed both how trainees performed and how they experienced the task.

## Discussion

In this section, we outline our key recommendations and reflect on the challenges of designing and implementing simulations that coach participants to engage with and apply metacognitive processes. We also consider emerging directions for research and innovation, including the integration of learning analytics and self-regulated learning, and discuss how these developments may shape the future design and evaluation of simulation-based education.

### Practical “top tips” for designing metacognitive simulation interventions

Key aspects to designing and implementing successful simulations for developing learners' metacognitive abilities include:

- Optimising communication to facilitate understanding; the utilization of visual aids and real-world examples are key to tapping into pre-existing understanding of metacognition (even if participants are not aware that they have experienced metacognitive feelings or behaviours before).
- Being aware of the literature; utilizing and adapting tested and accepted models streamlines the need to create a ‘new’ model from scratch, but equally being aware of positive applications, and their limitations, is key to selecting the optimal model for your own context.
- Simulation design; high levels of fidelity and realism may optimise transfer of learning to clinical practice. However, during the initial stages of metacognitive coaching, environmental fidelity and case complexity may need to be moderated to minimise cognitive overload and allow learners to focus on learning to apply the metacognitive model. Scenario complexity and fidelity can be progressively increased over repeated sessions to increasingly reflect real clinical practice.
- Collaboration; whether designing a metacognitive innovation or adapting an existing framework, utilizing expertise across academic and clinical areas is key. PERFORM was developed with the expert opinion of a sport psychologist, a supervisor with SRL expertise, and a PhD student with active acute patient experience. The surgical iteration of the model gained further expertise through recruitment of sabermetrics experts, educational directors and surgical trainees.

### Challenges, limitations, and considerations for educators

#### Metacognitive reflection

A key strategy is metacognitive reflection to make thinking visible by encouraging learners to explain their reasoning through think-aloud techniques, decision checkpoints, and reflective questioning. This helps identify assumptions, knowledge gaps, and clinical reasoning processes. Structured metacognitive reflection should also be embedded throughout the simulation cycle. For example, during pre-briefing, learners can set goals and anticipate challenges; during the scenario, they can monitor their performance and situational awareness; and during debriefing, they can evaluate decisions, emotions, and outcomes.

Debriefing is particularly important because it explores not only what learners did but why they acted as they did. Examining underlying assumptions and mental models promotes deeper understanding and supports metacognitive reflective practice. Educators should also encourage self-assessment and confidence calibration, helping learners to accurately match their perceived performance with actual outcomes so that they can strengthen the interaction between monitoring (judging one's performance) and control (adjusting strategies based on that judgment).

#### Importance of emotions in metacognitive reflection

Metacognitive reflection should include emotional awareness. Learners benefit from exploring how stress, uncertainty, confidence, and other emotions influenced their decision-making and communication. Developing awareness of these factors can enhance resilience, teamwork, and professional judgment by monitoring the learner's emotional responses and applying appropriate regulation strategies.

Despite its benefits, implementing metacognition in SBE can present challenges for educators. Learners often struggle with accurate self-assessment and may be overconfident or unaware of knowledge gaps. Time constraints can limit meaningful metacognitive reflection, while effective debriefing requires skilled facilitators capable of promoting psychological safety and exploring a learner's reasoning. Emotional discomfort, difficulties in measuring metacognitive processes and cognitive overload can further hinder implementation. Creating a learning environment which prioritises metacognitive reflection over clinical performance outcomes, not only focuses participants on the task at hand (and decreases cognitive overload by not trying to achieve ‘too much’ in one simulation session) but also quietly reinforces the importance placed on the self-regulation techniques. To achieve this in the original PERFORM study, and contrary to usual expectations for the junior doctor participants taking part in simulation, clinical markers and scores were purposefully not used and clinically focussed feedback was not delivered. Educational materials, protocols and signposting to additional resources could be provided to encourage self-directed study on particular clinical topics post-session, if required.

In summary, to maximise learning, educators should integrate metacognitive reflection throughout simulation activities, focus debriefing on reasoning and emotions, support self-monitoring, create psychologically safe environments, and provide repeated opportunities for metacognitive reflective practice and continuous improvement.

### The future: advancing simulation and expanding metacognitive models in global HPE

The next generation of SBE is expected to move beyond traditional post-event debriefing and self-reflection toward data-informed, technology-enhanced precision metacognitive feedback and real-time metacognitive support. Future systems may allow educators to measure metacognitive processes as they occur. There are increasing developments in artificial intelligence (AI), learning analytics and big data science, and self-regulated learning (SRL) microanalysis that can offer new opportunities to understand and develop a learner's metacognition with greater precision.

#### Learning analytics and adaptive metacognitive support

One of the most promising future developments is the integration of AI into simulation design and debriefing, especially with scenarios delivered through Virtual Reality (VR) and Augmented Reality (AR). AI can provide real-time understanding of a learner's metacognitive processes as they perform the simulation. For example, individual and team information-seeking behaviour, timing of clinical decisions, communication within teams and error recovery behaviours can be identified (16). A variety of physiological measures, such as heart rate, can also be collected to identify emotional responses (17). The data can be analysed and used to inform specific metacognitive feedback. Future AI systems may also function as a “metacognitive coach,” helping learners recognise gaps between their perceived and actual performance and supporting the development of more accurate metacognitive monitoring and regulation (18).

#### Self-regulated learning (SRL) microanalysis

SRL microanalysis is a modified ‘think aloud’ approach that can identify a learner's metacognitive processes during simulation by asking a series of specific questions before (goal setting), during (monitoring) and after (reflective evaluation) a clearly defined simulation task (19). This method can be readily integrated with the facilitator's routine feedback and is highly applicable to both low and high resource settings where AI may not be available (20).

## Future research

Future simulation research is expected to increasingly focus on AI-supported metacognitive coaching, the interaction between metacognition and emotion with the development of emotional monitoring and regulation during simulation, and team metacognition with a focus on collective monitoring and regulation.

In summary, future SBE is likely to extend beyond traditional debriefing into real-time, data-informed metacognitive support. AI, learning analytics, big data, and SRL microanalysis will deepen insight into a learner's metacognitive processes. In VR and AR, systems may track decisions, communication, and errors to deliver adaptive metacognitive feedback. Future tools could act as coaching systems, highlighting performance gaps while also exploring emotion, teamwork, and collective metacognition.

## Data Availability

The original contributions presented in the study are included in the article/supplementary Material, further inquiries can be directed to the corresponding author.

## References

[1] Grierson L Norman G Monteiro S Sibbald M Simulation-based Education and the Challenge of Transfer. In: Clinical Simulation edn. London: Academic Press. Chiniara G (2019). p. 115–27.

[2] Flavell JH. Metacognition and cognitive monitoring: a new area of cognitive-developmental inquiry. Am Psychol. (1979) 34(10):906–11. 10.1037/0003-066X.34.10.906

[3] Nelson TO. Metamemory: a theoretical framework and new findings. In: Bower GH, editor. Psychology of Learning and Motivation. San Diego (CA): Academic Press (1990) 26. p. 125–73. 10.1016/S0079-7421(08)60053-5

[4] Winne PH Azevedo R. Chapter 4: metacognition. In: Sawyer PK, editor. Cambridge Handbook of the Learning Sciences. Cambridge: Cambridge University Press (2014). p. 63–87.

[5] Cordeiro TL Santos LR Peixoto MA. Metacognition in clinical simulation: metacognitive events during briefing, simulated practice, and debriefing. Rev Bras Educ Med. (2025) 49. Available online at: https://www.scielo.br/j/rbem/a/n5vRftftkCYJ9GhMqjHPNRm/?format=pdf&lang=en

[6] Kolb DA. Experiential Learning: Experience as the Source of Learning and Development. Englewood Cliffs (NJ): Prentice-Hall (1984).

[7] Zimmerman BJ. Becoming a self-regulated learner: an overview. Theory Pract. (2002) 41(2):64–70. 10.1207/s15430421tip4102_2

[8] Schön DA. The Reflective Practitioner: How Professionals Think in Action. New York (NY): Basic Books (1983).

[9] Scharff L Draeger J Verpoorten D Devlin M Dvorakova LS Lodge JM. Exploring metacognition as a support for learning transfer. Teach Learn Inq. (2017) 5:1. 10.20343/teachlearninqu.5.1.6

[10] Efklides A. Interactions of metacognition with motivation and affect in self-regulated learning: the MASRL model. Educ Psychol. (2011) 46(1):6–25. 10.1080/00461520.2011.538645

[11] Efklides A Schwartz BL. Revisiting the metacognitive and affective model of self-regulated learning: origins, development, and future directions. Educ Psychol Rev. (2024) 36(2):61. 10.1007/s10648-024-09896-9

[12] Tappenden P. Problem structuring for health economic model development. In: Culyer AJ, editor. Encyclopedia of Health Economics. Amsterdam: Elsevier (2014), 168–179.

[13] Church HR Murdoch-Eaton D Sandars J. Using insights from sports psychology to improve recently qualified doctors’ self-efficacy while managing acutely unwell patients. Acad Med. (2021) 96(5):695–700. 10.1097/ACM.0000000000003809 33086231

[14] Efklides A. Metacognition: defining its facets and levels of functioning in relation to self-regulation and co-regulation. Eur Psychol. (2008) 13:277–87. 10.1027/1016-9040.13.4.277

[15] Howie EE Murray J Clarke R Totton N Peckham-Cooper A Church HR. Applying elite sports coaching routines to enhance cognitive load management and skill development in trainee surgeons: a pilot study. J Surg Educ. (2026) 83:103943. 10.1016/j.jsurg.2026.103943 41956835

[16] Echeverria V Zhao L Riordan A Milesi M Jin Y Abel S. Teamvision: an AI-powered learning analytics system for supporting reflection in team-based healthcare simulation. In: Proceedings of the CHI Conference on Human Factors in Computing Systems (CHI ‘25). New York (NY): Association for Computing Machinery (2025) 309, p. 1–22. 10.1145/3706598.3713395

[17] Elkin RL Beaubien JM Damaghi N Chang TP Kessler DO. Dynamic cognitive load assessment in virtual reality. Simul Gaming. (2024) 55(4):755–75. 10.1177/10468781241248821

[18] Buckley S Kos J Dass R Teng C Eaton K Zhang S Goel A A personalized AI coach to assist in self-directed learning. In: Wang Z Woodhead S Ananda M Basu Mallick D Sharpnack J Burstein J, editors. Proceedings of the Innovation and Responsibility in AI-Supported Education Workshop. Proceedings of Machine Learning Researc. 273. (2025). p. 221–29: Available online at: https://proceedings.mlr.press/v273/buckley25a.html

[19] Leggett H Sandars J Roberts T. Twelve tips on how to provide self-regulated learning (SRL) enhanced feedback on clinical performance. Med Teach. (2019) 41(2):147–51. 10.1080/0142159X.2017.1407868 29228830

[20] Cecilio-Fernandes D Carvalho DRS Sandars J Santos TM. Self-regulated learning microanalysis feedback for developing clinical skills: a pilot study. Med Teach. (2024) 46(10):1356–61. 10.1080/0142159X.2024.2308060 38295519

